# A medical records and data capture and management system for Lassa fever in Sierra Leone: Approach, implementation, and challenges

**DOI:** 10.1371/journal.pone.0214284

**Published:** 2019-03-28

**Authors:** Jeffrey G. Shaffer, John S. Schieffelin, Michael Gbakie, Foday Alhasan, Nicole B. Roberts, Augustine Goba, Jessica Randazzo, Mambu Momoh, Troy D. Moon, Lansana Kanneh, Danielle C. Levy, Rachel M. Podgorski, Jessica N. Hartnett, Matt L. Boisen, Luis M. Branco, Robert Samuels, Donald S. Grant, Robert F. Garry, the Viral Hemorrhagic Fever Consortium

**Affiliations:** 1 Department of Global Biostatistics and Data Science, School of Public Health and Tropical Medicine, Tulane University, New Orleans, Louisiana, United States of America; 2 Departments of Pediatrics and Internal Medicine, Sections of Pediatric & Adult Infectious Diseases, School of Medicine, Tulane University, New Orleans, Louisiana, United States of America; 3 Viral Hemorrhagic Fever Program, Kenema Government Hospital, Kenema, Sierra Leone; 4 Ministry of Health and Sanitation, Freetown, Sierra Leone; 5 Department of Microbiology and Immunology, Tulane University, New Orleans, Louisiana, United States of America; 6 Vanderbilt University Institute for Global Health, Nashville, Tennessee, United States of America; 7 Zalgen Labs, LLC, Germantown, Maryland, United States of America; Institute of Tropical Medicine Antwerp, BELGIUM

## Abstract

Situated in southeastern Sierra Leone, Kenema Government Hospital (KGH) maintains one of the world’s only Lassa fever isolation wards and was a strategic Ebola virus disease (EVD) treatment facility during the 2014 EVD outbreak. Since 2006, the Viral Hemorrhagic Fever Consortium (VHFC) has carried out research activities at KGH, capturing clinical and laboratory data for suspected cases of Lassa fever. Here we describe the approach, progress, and challenges in designing and maintaining a data capture and management system (DCMS) at KGH to assist infectious disease researchers in building and sustaining DCMS in low-resource environments. Results on screening patterns and case-fatality rates are provided to illustrate the context and scope of the DCMS covered in this study. A medical records system and DCMS was designed and implemented between 2010 and 2016 linking historical and prospective Lassa fever data sources across KGH Lassa fever units and its peripheral health units. Data were captured using a case report form (CRF) system, enzyme-linked immunosorbent assay (ELISA) plate readers, polymerase chain reaction (PCR) machines, blood chemistry analyzers, and data auditing procedures. Between 2008 and 2016, blood samples for 4,229 suspected Lassa fever cases were screened at KGH, ranging from 219 samples in 2008 to a peak of 760 samples in 2011. Lassa fever case-fatality rates before and following the Ebola outbreak were 65.5% (148/226) and 89.5% (17/19), respectively, suggesting that fewer, but more seriously ill subjects with Lassa fever presented to KGH following the 2014 EVD outbreak (p = .040). DCMS challenges included weak specificity of the Lassa fever suspected case definition, limited capture of patient survival outcome data, internet costs, lapses in internet connectivity, low bandwidth, equipment and software maintenance, lack of computer teaching laboratories, and workload fluctuations due to variable screening activity. DCMS are the backbone of international research efforts and additional literature is needed on the topic for establishing benchmarks and driving goal-based approaches for its advancement in developing countries.

## Background

**Fig 1:**
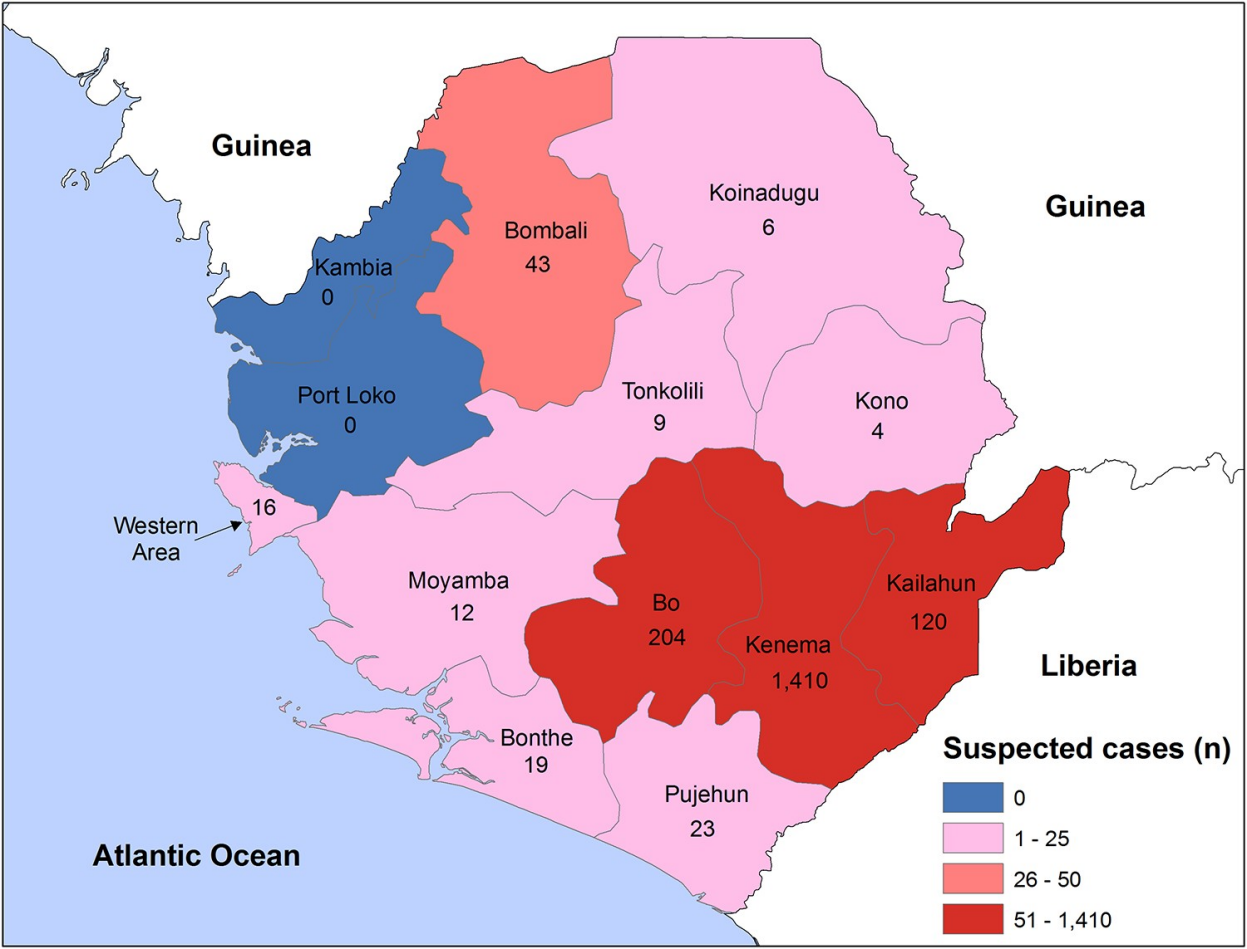
District of residence for suspected Lassa fever case screenings at Kenema Government Hospital, 2008–2016. District of residence was available for 44.1% (1,866/4,229) of the screened study subjects. Regions with the highest observed Lassa fever screenings were the Districts of Kenema (n = 1,410), Bo (n = 204), and Kailahun (n = 120).

Lassa fever (also referred to here as Lassa) is a hemorrhagic illness that is transmitted to humans primarily through contact with rodent excreta. The disease was first detected in 1969 in Lassa, Nigeria, and stems from an Old World arenavirus known as Lassa virus [1, 2]. Lassa is currently found in sub-Saharan West Africa and is endemic to parts of Sierra Leone, Liberia, Guinea, and Nigeria. The disease is distinctive from other arenaviruses in that human-to-human transmission may occur through infected blood or bodily fluids [3–5]. There is currently no vaccine available for Lassa, and it accounts for between 100,000 and 300,000 infections and 5,000 deaths annually [6, 7]. Lassa infections most commonly arise in poor, rural areas, partly due to insufficient food storage practices and increased interaction between humans and animals found in such areas. Some of the world’s highest observed Lassa fever infection rates are found in Sierra Leone’s Kenema District that is located in its Eastern Province near its south Liberian border (Fig 1; [8, 9]).

Kenema Government Hospital (KGH) lies in the central part of Kenema District and maintains Sierra Leone’s only Lassa fever treatment center and isolation unit. The hospital maintains a biosafety level 3 (BSL-3) laboratory where blood samples for suspected Lassa cases are routinely screened using rapid, enzyme-linked immunosorbent assay (ELISA), and polymerase chain reaction (PCR) diagnostics. KGH was a central screening, treatment, and isolation facility for Ebola virus disease (EVD) during the 2014–2016 EVD epidemic [8, 10, 11].

Data systems in Sierra Leone are notoriously weak. From 1991–2002, Sierra Leone was ravaged by civil conflict, destroying the country’s scant infrastructure and health systems [12]. It is estimated that electricity reaches less than 10 percent of Sierra Leone’s population, directly impacting computer literacy, capacity for training and education, and the ability to build contemporary data systems [13]. In 2006, Sierra Leone’s data systems were deemed inadequate according to virtually all criteria assessed by the Health Metrics Network [14, 15]. Also in 2006, Sierra Leone was among several countries that did not respond to a survey by the World Health Organization (WHO) on the state of their national health research data systems [16, 17]. In 2007, Sierra Leone maintained internet penetration rates of 0.2%, or 10,000 users out of a population of 6,015,417 persons [16–19]. Effective coordination of health information has been described as particularly weak throughout Sierra Leone, often resulting in duplication and gaps in the collection, reporting, use, and management of data [20]. Dawson et al. (2011) described the development of a medical records system at a hospital in Freetown, Sierra Leone and capturing data on subjects following hospital discharge as one of their key challenges [21]. Frieden and Damon (2015) discussed the overwhelming burden of the 2014 Ebola outbreak on the KGH data systems and acknowledged support provided during the outbreak by the Centers for Disease Control and Prevention (CDC) for carrying out data management and geographic information systems (GIS) activities for tracking and evaluating disease trends [22]. Owada et al. (2016) noted the lack of processes in reporting confirmed EVD cases in holding, treatment, and community care centers as primary data management challenges during the 2014 Ebola outbreak [23].

While data capture and management system (DCMS) platforms are primarily paper-based in Sierra Leone, incremental strategies for moving toward electronic DCMS processes is widely recommended [21]. Recent efforts to improve DCMS in Sierra Leone include the implementation of open-source modular electronic health platforms such as Open Medical Record System (OpenMRS), national health data management systems such as Health Information System Programme’s health information software, and mobile electronic medical records systems such as Ebola EMR [24–27]).

Since 2006, researchers with the Viral Hemorrhagic Fever Consortium (VHFC) have carried out a host of research projects through coordinated efforts with the National Institutes of Health (NIH), the Sierra Leonean Ministry of Health and Sanitation (MOHS), and KGH. These efforts have led to considerable developments in laboratory and clinical capacity, particularly in the area of diagnostic testing. A significant byproduct of these efforts includes a Lassa fever DCMS at KGH with the dual purpose of serving hospital and research responsibilities that has played an instrumental role in research advancements on Lassa fever and EVD [11, 28]. Here we focus on the development and progress of a Lassa fever DCMS at KGH between 2010 and 2016 developed through a collaboration between Tulane University and KGH. Selected results on Lassa fever screening patterns and case-fatality rates are provided in an effort to provide context and illustrate the scope of the DCMS.

## Materials and methods

### Ethics statement

This project was approved by the Tulane University Institutional Review Board and the Sierra Leone Ethics Committee. Blood samples for study subjects were collected at local peripheral health units or KGH based on the suspicion of Lassa fever. Patients presenting to KGH were cared for or treated by trained KGH personnel [4]. Written informed consent was obtained for all subjects at least 18 years of age, and written parental permission was obtained for all subjects under 18 years of age. Subjects 7 to 17 years of age also provided an affirmative agreement to participate in the study through an assent process with age-appropriate language.

### Study site

Kenema Government Hospital (KGH) is situated in southeastern Sierra Leone in Kenema District approximately 30 miles (48 kilometers) from its Liberian border. KGH maintains one of the world’s only Lassa fever isolation wards with full-time staff and clinicians. Samples for suspected Lassa fever cases are screened at the KGH Lassa laboratory (a BSL-3 facility), which serves as the primary screening facility for the entire country of Sierra Leone and occasionally for neighboring parts of Liberia and Guinea. Screening occurs through passive case detection (PCD) for suspected Lassa cases and active case detection (ACD) following case confirmation through clinical and diagnostic testing.

### Electricity and internet sources

The DCMS was developed under extreme resource constraints, including limitations in electricity and internet functionality. The electrical power system at the KGH Lassa Ward and Laboratory included the following power sources in successive order: 1) grid-based power systems (known as *town power*); 2) solar power systems through roof-mounted solar panels; and 3) gasoline electric generator power systems. Town power was only partially available at KGH outside of daily operating hours, so essential laboratory equipment and refrigerators commonly required joint reliance on solar and electrical generator power sources. The solar power source was usually inadequate for independently supporting the demands for the KGH Lassa Ward and was complemented with gasoline electrical generator power, particularly in the evenings or in the absence of sunlight.

Through 2011, internet availability for cloud-based data capture and file sharing was typically unavailable. In 2012, satellite internet connectivity was introduced to the KGH Lassa Ward via the Constellation Network Satellite Internet System (Constellation, Inc, Traverse City, MI). Following the 2014 Ebola outbreak, internet connectivity was acquired through commercial vendors and governmental sources based in Sierra Leone’s capital city of Freetown.

### Patient triaging and data flow

The DCMS was centrally situated at KGH. The system was integrated into the KGH Lassa Ward and Laboratory units for accommodating suspected cases of Lassa fever presenting to KGH or one of its local peripheral health units. Occasionally, health units or hospitals in Liberia or Guinea transported blood samples or subjects to KGH for Lassa fever screening or patient care. Patient triaging was based on the suspected case definition for Lassa fever shown in Table 1.

**Table 1:** Lassa fever suspected case definition.

Known exposure to a suspected case of Lassa fever or	
Temperature over 38°C for less than three weeks and	
Absence of signs of local inflammation and	
Two major signs or one major sign and two minor signs:	
**Major signs**	**Minor signs**
Bleeding	Headache
Neck or facial swelling	Sore throat
Conjunctivitis or sub-conjunctival hemorrhage	Vomiting
Spontaneous abortion	Diffuse abdominal pain or tenderness
Petechial or hemorrhagic rash	Retrosternal or chest pain
New onset of tinnitus or altered hearing	Cough
Persistent hypotension	Diarrhea
Absence of clinical response after 48 hours to anti-malarial or broad spectrum antibiotic therapy	Generalized myalgia or arthralgia
	Profuse weakness

Modifed from Khan et al. (2008; [4]) and reproduced from Shaffer et al. (2014; [28]).

**Fig 2:**
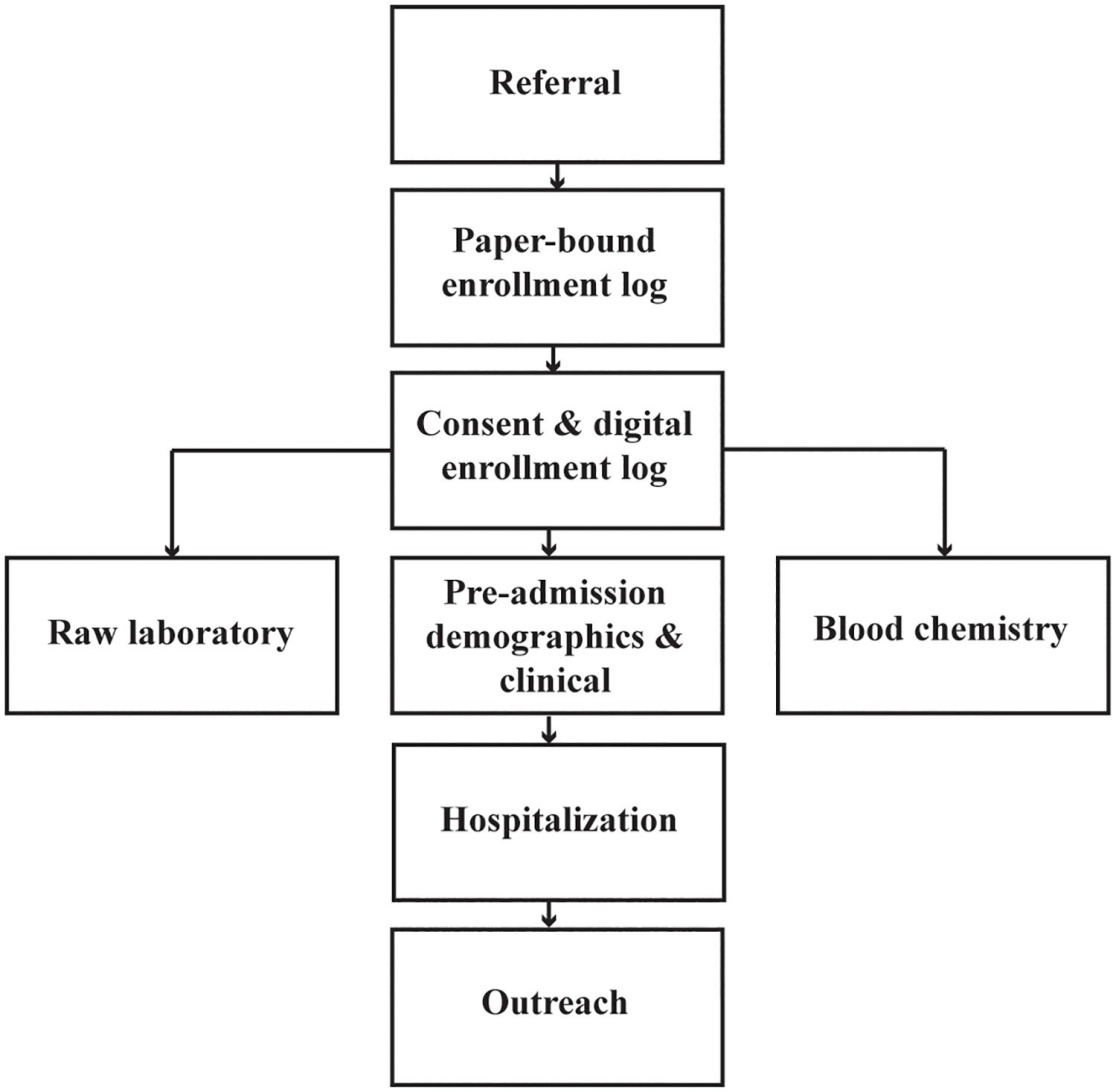
Data flow at Kenema Government Hospital Lassa Ward. The conceptual framework for data flow processes spanned patient triaging, pre-admission clinical evaluation, laboratory testing, hospitalization, and case finding.

On presentation, subjects interested in participating in the study provided written informed consent, underwent a clinical evaluation, and provided a blood sample for Lassa fever and blood chemistry screening. Suspected Lassa fever cases presenting to peripheral health units provided blood samples at the peripheral health units, and these samples were then transported to KGH for Lassa fever screening. Those subjects testing positive for Lassa fever were transported to KGH for additional screening and further clinical evaluation. Blood samples were screened for Lassa using rapid diagnostic tests (RDTs), ELISA (antigen [Ag], immunoglobin M [IgM], and immunoglobin G [IgG]), and PCR testing methods at the KGH Lassa laboratory. Hospital admission criteria were based on the Lassa fever suspected case definition (Table 1), diagnostic test results, and a full clinical evaluation [28]. Admitted subjects were isolated at the KGH Lassa Isolation Ward, and clinical assessments and laboratory tests were repeated during hospitalization to monitor patient recovery. Case finding operations were set up to identify, locate, and screen close family and household members that were in close contact (referred to as “contacts”) with the confirmed cases. Those contacts meeting the suspected case definition for Lassa were then considered as suspected Lassa cases and transported to KGH for Lassa screening and patient care. The conceptual framework of the KGH Lassa fever data flow, spanning patient triaging, pre-admission clinical evaluation, laboratory testing, hospitalization, and case finding is shown in Fig 2.

### Data center and personnel

A data center (referred to here as the Lassa Data Center) was strategically set up outside of (but in close proximity to) the KGH Lassa Ward and Laboratory to facilitate the completion, signature, and review of clinical forms and questionnaires while minimizing the transfer of paper materials between hospital units. DCMS operations were carried out at the KGH Lassa Ward, Lassa Laboratory, and Lassa Data Center. DCMS personnel included hospital intake workers, nursing staff, a laboratory director, laboratory technicians, an on-site data manager, a computer technician, outreach personnel, an on-site physician, an off-site physician, an off-site biostatistician, and off-site research assistants.

### Case report form (CRF) development

Data collection was carried out using paper-bound and digital enrollment logs; a suite of clinical questionnaires and forms (referred to here as case report forms [CRFs]); and laboratory equipment, including PCR (thermal cycler) machines, ELISA plate readers, and Piccolo Xpress blood chemistry analyzers (Abaxis Global Diagnostics, Union City, CA). All laboratory data was maintained in both paper-bound laboratory notebooks and electronic format. Data classes and sources covered clinical and laboratory data before, during, and following hospitalization (Table 2).

**Table 2:** Data classes and capture sources for Lassa fever at Kenema Government Hospital.

Data class	Data capture source
1. Referral	Referral and clinical evaluation forms from peripheral health units
2. Enrollment	Enrollment and patient consent log books
3. Pre-admission clinical	Pre-admission clinical evaluation CRFs
4. Raw laboratory diagnostic	Paper-bound laboratory notebooks and electronic data files generated by ELISA plate readers and PCR (thermal recycler) machines
5. Summary laboratory diagnostic	Laboratory results forms summarizing raw laboratory results for hospital and patient use
6. Blood chemistry	Electronic data files generated by Piccolo Xpress blood chemistry panel analyzers
7. Hospitalization	Post-admission clinical CRFs
8. Outreach	Case contact tracing CRFs (for active case detection)
9. Audit	Patient chart reviews, paper-bound clinical and laboratory notebooks, CRF and electronic file inventory audits

ELISA = enzyme-linked immunosorbent assay; CRF = case report form; PCR = polymerase chain reaction.

### Medical charts and clinical data capture (Data classes 1–3)

Clinical data capture covered data sources from pre- and post-hospitalization clinical sources using a coordinated set of log books and CRFs. In response to an on-site clinical trial in 2012, a suite of CRFs was developed as the prototype for patient medical charts for suspected cases of Lassa fever. CRFs were classified according to stage of patient care (referral, pre-admission, hospitalization, and post-discharge), and a lettering system was used to imprint the classifications on the CRFs. Medical charts were maintained in manila folders and secure file cabinets, which was novel to the KGH Lassa Ward at the beginning of this study. Prior to the use of file cabinets, CRFs were occasionally burned or destroyed due to contamination concerns. The CRFs were developed by building on earlier CRF versions to facilitate the transition and maintain familiarity by hospital data collection personnel. CRFs were printed using single-sided printing to ensure that the back sides of pages were not overlooked during completion. Most of the CRF items provided closed-ended responses by including responses such as “unspecified” or “unknown.” For those cases where appropriate closed-ended response options were not listed, data collection personnel were instructed to log responses into open-ended comments fields. Common open-ended responses were then identified and used to consider new closed-ended responses on subsequent CRF versions. All of the CRFs required doctor authentication and signature. Editable versions of CRFs were maintained by limited personnel to prevent unauthorized changes.

### Laboratory data capture (Data classes 4–6)

Patient blood samples were tested for Lassa using Ag, IgM, and IgG ELISA; polymerase chain reaction (PCR); and rapid lateral-flow immunoassay (LFI) diagnostics. Data for the ELISA techniques were generated using BioTek microplate readers (BioTek, Winsooki, VT) with the Gen5 Microplate Data Collection and Analysis Software (BioTek, Winooski, VT). ELISA data were exported into individual Microsoft Excel (Microsoft Corporation, Redmond, WA) workbooks, and a suite of Visual Basic (Microsoft Corporation, Redmond, WA) macros was used to consolidate and organize these workbooks into individual summary files. The Visual Basic application was chosen for its compatibility with other software applications and its ease of use within the Microsoft Office framework. Laboratory staff were trained on running the macros and were responsible for uploading the summary workbooks to a shared, cloud-based location on a monthly basis. Beginning in January 2012, patient blood samples were also analyzed using Piccolo Xpress (Abraxis, Union City, CA) blood chemistry analyzers. An electronic template equipped with automatic range and logic checks was developed for restructuring blood chemistry panel data files into rectangular data sets.

### Post-hospitalization data capture (Data classes 7–8)

Post-hospitalization CRFs focused on case investigation and contact tracing activities, capturing data at the individual level (e.g., age, gender) and household level (e.g., age, gender, and dwelling structure, food storage practices). Closeout forms were included with the post-admission CRFs to verify that outreach activities were carried out at scheduled time intervals. Following patient discharge, CRFs in the medical charts were scanned using Fujitsu ScanSnap iX500 (San Jose, CA) automatic feed scanners and optical character recognition (OCR). The Adobe Acrobat Redact Tool (Professional Version 11, Adobe Inc., San Jose, CA) was used to redact personally identifiable data from the CRFs. The CRFs were classified according to individual study subject identification numbers and scanned into single Portable Document Format (PDF) files named according to their corresponding study identification numbers. An optimization process was used to reduce the size of the scanned charts, often reducing file sizes from the order of megabytes to bytes. The filename system was standardized according to the study identification numbers suffixed with letters indicating whether redaction processes were complete.

### Data audits and audit data capture (Data class 9)

The DCMS was designed to capture data for key study outcomes from multiple sources to provide a mechanism for cross-verification and provide secondary data sources to account for missing data. Data were audited over biannual, KGH site visits by comparing data files against source documents. For key study variables such as patient survival outcome and dates of admission and discharge, all data were audited against paper-bound source records. For laboratory data groups, sets of random electronic records were chosen and verified against paper-bound laboratory notebooks. Additionally, data audits were carried out remotely by comparing the numbers of scanned CRFs against study identification number counts from enrollment logs to determine scanning backlogs and identify and locate any missing medical charts. Audit data were maintained in separate data files to retain audit trails and were deemed as higher orders of validity than data captured from other sources. The data audits regularly revealed modest gaps in capturing survival outcome data, high levels of correspondence between electronic laboratory data and their associated paper-bound laboratory notebooks, and lag times of up to two months for scanning and uploading CRFs to shared archives.

### Database design

**Fig 3:**
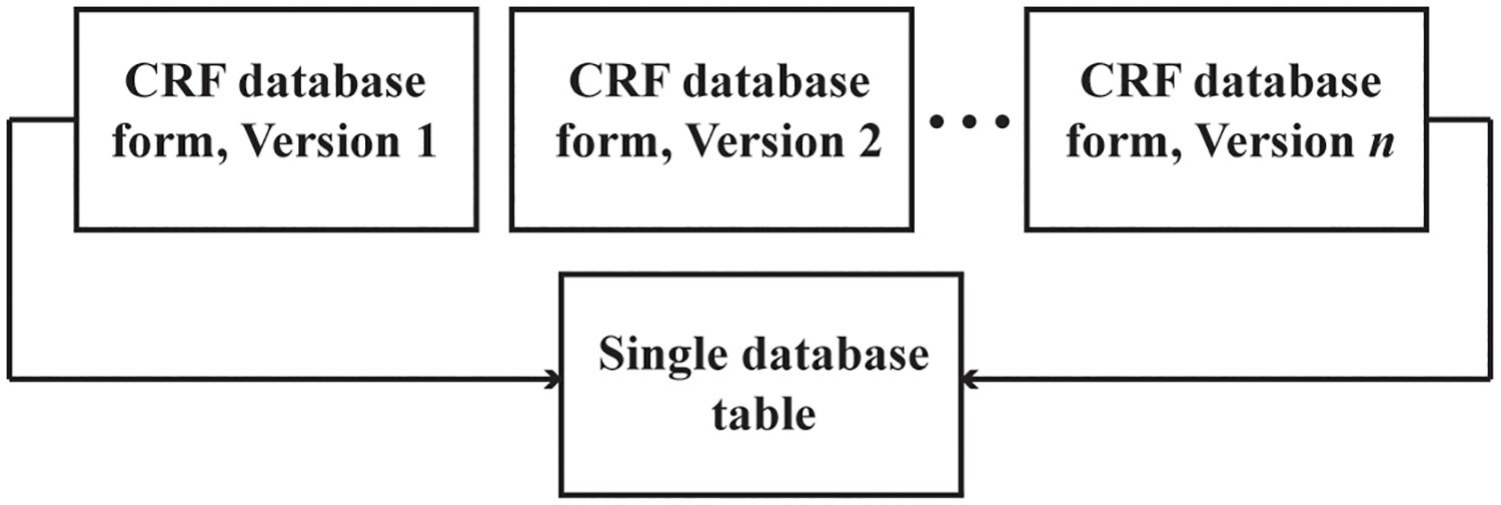
Database structure for multiple database forms linked to a single database table. To account for CRF versioning, the database forms for multiple versions of a specific CRF were linked to a single underlying database table.

A relational database was developed using the Microsoft Access (Versions 2007, 2010, and 2013; Microsoft, Redmond, WA) database management system. The database included a suite of database forms bounded to rectangular data tables and automated summary reports. Data entry was carried out by logging data from the CRFs into the database forms, and the bounded database tables were used for data management. The database was equipped with lookup queries (for automatically populating data captured on multiple forms, such as patient age and gender), validation rules, and input masks. Date values were formatted with three-letter month abbreviations to preclude day and month transposition errors. To account for CRF versioning, a database form was developed for each specific CRF version and linked to a single underlying database table. This bounding process is illustrated in Fig 3.

Those fields removed from CRFs as a result through versioning were retained in their database tables to maintain data for any single CRF in a single data set. Validation of the database forms was performed by matching logged data against their corresponding data tables. This process was based on inputs of 10 records for each database form, and database forms were considered as validated once exact correspondence was observed between the database forms and the underlying data tables. No personally identifiable data was logged into the database, and it was maintained on password-protected computers. Database access was restricted to key project personnel or personnel with data management or data entry responsibilities.

### Data sharing and backup

Laboratory data groups such as ELISA, PCR, and blood chemistries were generated in electronic formats and uploaded to cloud-based archives on a monthly basis. Hardwired servers were not available at KGH, so file sharing was largely dependent on internet connectivity using cloud-based file sharing applications. In 2010, a Microsoft SharePoint (Microsoft Corporation, Redmond, WA) site was developed and piloted, but the bandwidth at the KGH was not conducive to its continued use. Later in 2010, file sharing employed the DropBox application (DropBox, Inc., San Francisco, CA) and subsequently the joint use of the DropBox and Box (Box, Inc., Redwood City, CA) applications. The Box application was preferred here because of its university license agreement and its Health Insurance Portability and Accountability Act (HIPPA) compliance. Neither of these features were available for the DropBox application during the course of this study. Data backup was initially performed using external hard drives at the KGH site, but the process later transitioned to a cloud-based file uploading approach using a combination of the DropBox and Box applications.

### Data management, verification, and curation

Redacted patient charts were posted to a shared *Box* folder and shared among key project personnel. Data were logged by a data manager at KGH using computers equipped with uninterruptible power supply (UPS) devices to guard against power failure during data entry, management, and backup. Data were independently logged at the Tulane University site using the scanned, archived CRFs stored in the shared *Box* folder. Data discrepancies were determined through mismatch queries using the SAS System (SAS Institute, Cary, NC) or Microsoft Access and served as the basis for data audits at KGH. All data sources were linked into a single master data set based on patient and blood sample identifiers using the SAS System.

### Determination of serostatus

Raw laboratory data was considered valid only if each of its two optical density values were nonnegative (for both the study subject blood samples and their associated positive and negative control samples) and the optical density values for the positive control samples were greater than those for their corresponding negative control samples. All serostatus results for this work were based on the initial blood draw. In the event that multiple tests were carried out on the initial blood draw, the first test was chosen to represent the patient sample. Test positivity for Ag (IgM) ELISA was determined according to 2.5 (3.5) standard deviations above the average of the negative control values collected in a specific year of blood draws. These break points were calculated by year of blood draw to control for environmental and temporal differences and technological advancements at the KGH Lassa laboratory.

## Results

**Fig 4:**
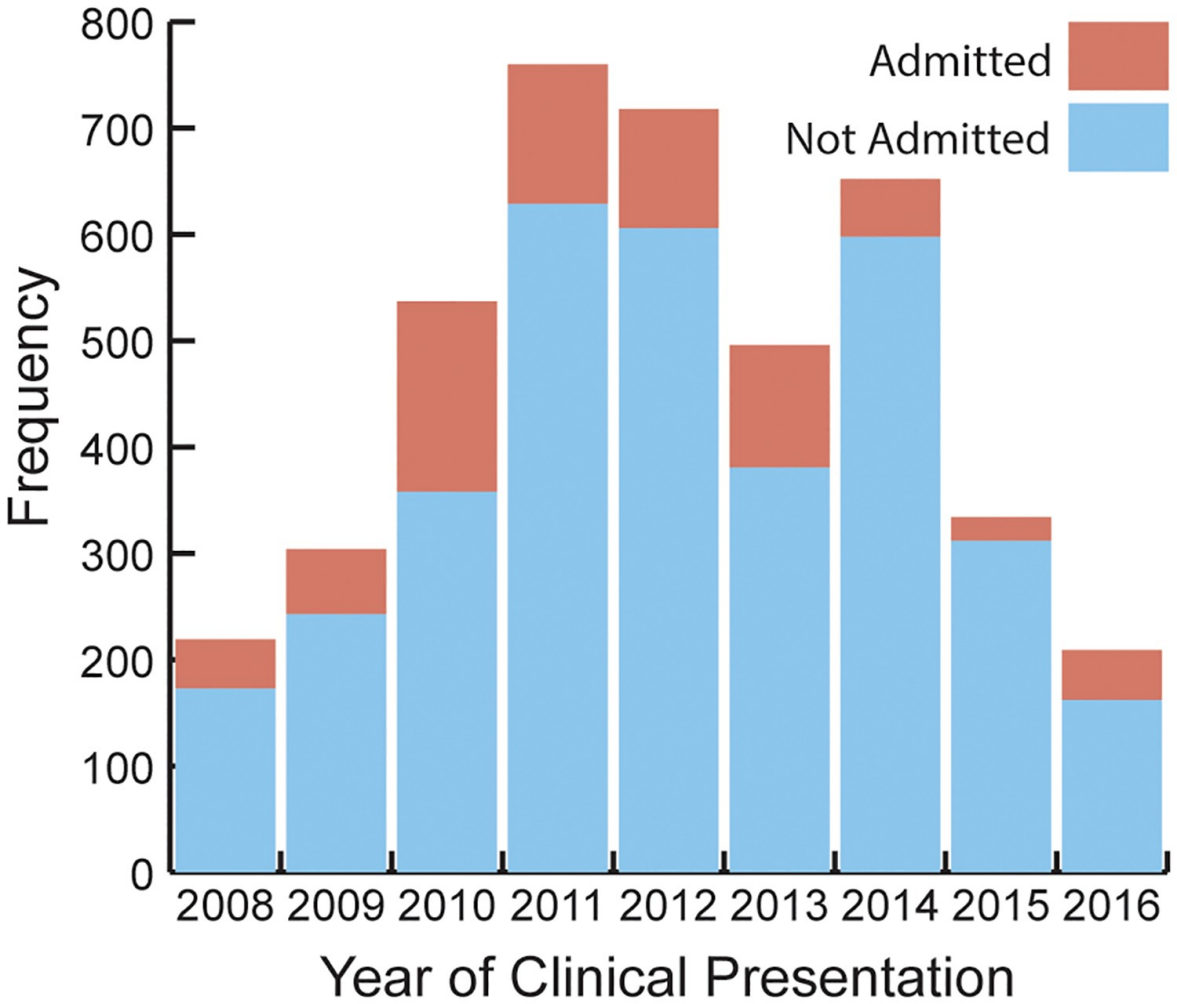
Distribution of blood samples screened for Lassa fever at Kenema Government Hospital, 2008–2016. Screenings for subjects not admitted to KGH primarily resulted from samples provided to KGH by one of its peripheral health units without subject presentation or subjects who died prior to arrival at KGH.

Between January 2008 and December 2016, blood samples for 4,229 subjects were screened at KGH for Lassa fever, resulting in 767 hospital admissions (Fig 4).

Screenings for subjects not admitted to KGH were primarily due to samples submitted to KGH from one of its peripheral health units without subject presentation or subjects who died prior to arrival at KGH. The peak suspected case counts in 2011 were partially due to increased screenings for case contacts (data not shown). This peak may also reflect changes in health seeking behaviors, increased Lassa fever awareness, and more aggressive screening practices. Serostatus results are shown by survival outcome at hospital discharge (or following consultation for subjects not admitted to KGH) in Table 3.

**Table 3:** Lassa fever serostatus results, Kenema Government Hospital, 2008–2016.

Survival outcome at discharge^b^	Lassa Fever Serostatus^a^			*p* value^c^
	Ag+/IgM+- (n = 389)	Ag-/IgM+ (n = 885)	Ag-/IgM- (n = 2,955)	
Died	165 (42)	65 (7)	114 (4)	< .001
Discharged	80 (21)	199 (23)	237 (8)	
Unknown	144 (37)	621 (70)	2,604 (88)	

Individual data provided as supporting information in S1 Table.

^a^Ag+/IgM+- = Acute Lassa fever exposure (samples testing positive according to Ag ELISA); Ag-/IgM+ = Recent Lassa fever exposure (samples testing negative according to Ag ELISA and positive according to IgM ELISA); Ag-/IgM- = Absence of recent Lassa fever exposure (samples testing negative according to Ag and IgM ELISA).

^b^Survival outcome measured at hospital discharge (or following initial consultation for subjects not admitted to KGH). Observations for subjects transferred from the KGH Lassa Ward to other KGH units were included in the “Unknown” survival outcome category.

^c^Fisher’s Exact Test comparing serostatus groups for general differences.

The largest proportion of samples was observed for subjects testing negative for Lassa fever, which may be a result of the weak specificity of the Lassa fever suspected case definition or variable triaging practices from KGH peripheral health units. The Lassa fever suspected case definition shares many of the signs and symptoms with suspected case definitions for competing febrile illnesses, including malaria and EVD. Malaria testing was not routinely carried out in this study, and thus it is not possible to describe its impact here. While fewer than 100 confirmed Lassa cases were observed annually (389 between 2008 and 2016), these estimates likely (and perhaps grossly) underestimate the actual Lassa fever case counts in Sierra Leone due to the self-presentation bias of the screenings. The observed case fatality rates (CFR) restricted to died or discharge classifications was 67.3% (165/245). A more conservative CFR estimate assuming that subjects with an unknown survival outcome ultimately survived was 42.4% (165/389). The fatality rates for subjects testing negative for Lassa fever was 32.5% (114/351), suggesting that presenting patients were generally very ill regardless of their Lassa fever serostatus. It is worth noting, however, that survival outcomes were usually unobserved for subjects testing negative for Lassa fever.

Survival outcome data was only available for 20.3% (860/4,229) of subjects, and unknown patient survival outcomes stemmed from several factors. First, patient triaging practices were variable across the peripheral health units. Also, subjects that tested positive for Lassa did not always present to KGH, which included patients dying prior to arrival at KGH or patients discharged from peripheral health units against clinical advice. Further, patient survival outcomes occurred at different time points during hospitalization and were not always prompted by specific intake or discharge forms for capturing survival outcome data. Finally, the lack of an integrated data system across all of the KGH units (such as its Maternity Ward) rendered the ultimate outcomes of subjects transferred to other parts of the hospital as unknown.

### Impact of the 2014 Ebola outbreak

The first case of EVD in Sierra Leone was detected at the KGH Lassa Laboratory on May 24, 2014 [8]. The influx of suspected EVD cases had direct implications on the Lassa fever DCMS. First, the DCMS was designed to accommodate data for suspected cases of Lassa fever. During the outbreak, patient triaging became increasingly challenging as the suspected case definitions for Lassa fever and EVD include similar or overlapping criteria. Also, the KGH Lassa Ward was among only several locations in Sierra Leone that maintained patient isolation and laboratory screening capacity, and thus incoming suspected cases of EVD were triaged there for diagnosis and treatment. During the initial stages of the outbreak, the Lassa fever CRFs and laboratory screening and testing processes were partially adapted to capture data on suspected EVD cases [11]. The influx of EVD presentations resulted in substantial declines in the number of suspected Lassa cases presenting to KGH, which may be attributable to anxiety about the deadly and highly contagious nature of Ebola, particularly at health care centers. While the end of the outbreak was officially declared by the World Health Organization on November 7, 2015, presentations for Lassa or any illness during or following the Ebola outbreak are likely underestimated (and in many cases significantly underestimated) due to changes in health seeking behaviors [29]. This phenomenon illustrates the importance of enhanced outreach operations during unanticipated outbreaks of highly contagious illnesses. The characterization of suspected Lassa fever cases before and following the Ebola outbreak is shown in Table 4.

**Table 4:** Characteristics of suspected Lassa fever cases before and after the 2014 Ebola virus disease outbreak, Kenema Government Hospital, 2008–2016.

Characteristic	Pre-Ebola n = 3,192	Post-Ebola n = 1,037	*p* value^d^
Admission status, *n* (%)			
Admitted	694 (22)	73 (7)	< .001
Not admitted	2498 (78)	964 (93)	
Survival outcome, *n* (%)^a^			
Died	313 (39)	31 (61)	.003
Discharged	496 (61)	20 (39)	
Serostatus, *n* (%)^b^			
Ag+/IgM+-	314 (10)	75 (7)	< .001
Ag-/IgM+	699 (22)	186 (18)	
Ag-/IgM-	2,179 (68)	776 (75)	
Serostatus by survival outcome, *n* (%)^b^^,^^c^			
Ag+/IgM+-			
Died	148 (65)	17 (89)	.040
Discharged	78 (35)	2 (11)	
Ag-/IgM+			
Died	62 (25)	3 (18)	.771
Discharged	185 (75)	14 (82)	
Ag-/IgM-			
Died	103 (31)	11 (73)	.001
Discharged	233 (69)	4 (27)	

Corresponding individual data provided as supporting information in S1 Table. Pre- and post-Ebola classifications defined according to the first diagnosed case of Ebola virus disease at KGH on May 24, 2014 [8].

^a^Survival outcome measured at hospital discharge (for admitted subjects) or following clinical evaluation (for non-admitted subjects). Survival outcomes were missing for 2,383 subjects in pre-Ebola time period and 986 subjects in post-Ebola time period.

^b^Ag+/IgM+- = Acute Lassa fever exposure (samples testing positive according to Ag ELISA); Ag-/IgM+ = Recent Lassa fever exposure (samples testing negative according to Ag ELISA and positive according to IgM ELISA); Ag-/IgM- = Absence of recent Lassa fever exposure (samples testing negative according to Ag and IgM ELISA).

^c^Frequencies of missing survival outcomes by serostatus group in pre-Ebola and post-Ebola time periods, respectively were: Ag+/IgM+- (88 and 56); Ag-/IgM+ (452 and 169); and Ag-/IgM- (1,843 and 761).

^d^Fisher’s Exact Test comparing characteristics between pre-and post-Ebola time periods. *p* values for comparisons involving patient survival outcome were calculated excluding unknown survival outcomes.

Overall fatality rates (regardless of Lassa fever serostatus) before the Ebola outbreak were 60.7% [31/51], which was significantly higher than the those prior to the outbreak (38.7% [313/809]; p = .003). Lassa fever case-fatality rates before and following the Ebola outbreak were 65.5% (148/226) and 89.5% (17/19), respectively, suggesting that fewer, but more seriously ill subjects with recent Lassa fever exposure presented to KGH following the outbreak (p = .040). These results suggest that illness severity generally increased among subjects presenting to KGH following the Ebola outbreak, which may be related to changes in health seeking behaviors. The proportion of subjects admitted to the KGH Lassa Ward prior to the outbreak was 21.7% (694/3,192) compared to just 7.0% (73/1,037) following the outbreak, and this decline was statistically significant (p < .001). The proportion of Ag-/IgM- subjects significantly increased following the outbreak (68.3% [2,179/3,192] before the outbreak versus 74.8% [776/1,037] following the outbreak; p < .001). Together, these results may indicate less aggressive screening practices or changes in patient triaging practices at peripheral health units following the Ebola outbreak. The implications of these findings on the Lassa fever DCMS were an increased focus on data capture for more seriously ill subjects presenting to KGH with non-Lassa febrile illnesses.

### DCMS strengths

The DCMS solutions here jointly served hospital and research purposes, maintained low operating costs, yielded primary and secondary data sources, and provided sustainability within the KGH framework. Software solutions for the DCMS were inexpensive or freely available. The DCMS captured data from multiple sources for key study variables such as patient survival outcome, which provided alternative data sources where data were missing through primary sources. An on-site clinical trial for a Lassa fever rapid testing diagnostic test in 2012 provided perspective for standardizing and strengthening the DCMS CRF system and laboratory processes. The notion of developing a DCMS in the sense of a clinical trial is detailed by Prokscha (2012; [30]).

A notable strength of the DCMS was the outstanding interpersonal skills and work ethic of the KGH personnel. It was not uncommon for lead physicians or hospital administrators to be directly involved data-related matters. Hospital intake workers and nursing staff were meticulous in collecting data and ensuring the completion of CRFs in their entirety. The laboratory staff provided considerable support in DCMS operations, which was particularly important in coordinating laboratory data management activities with data management and hospital personnel. The DCMS personnel showed exemplary courage and valor carrying out their job responsibilities in the face of the deadly Ebola outbreak and captured valuable clinical and diagnostic data, making possible one of the largest clinical studies on Ebola to date [11].

### DCMS challenges

The DCMS challenges may be broadly considered in terms of data linkage and integration, CRF structure, software availability, internet functionality, and electrical power sources. These challenges are further classified and detailed below.

#### Linkage with external data systems

The Lassa fever DCMS operated as an independent system with limited linkage to outside KGH units (those units not involved with Lassa fever responsibilities) and outside surveillance networks. Incorporating data for suspected cases of Lassa fever transferred to or from outside KGH units would have resulted in more complete data sources. An example of this limitation was described earlier where survival outcomes for subjects transferred from the KGH Lassa Ward to other KGH units were considered as “unknown.”

#### Automation of data linkage

The DCMS was used to capture data over multiple sources, and data linkage and integration was not fully automated. The lack of fully automated DCMS processes was partly due to the use of paper-based CRFs, the large number of data sources, and the multi-site (KGH and Tulane University) approach for carrying out data quality control procedures.

#### Limited data management personnel

KGH personnel fully designated for DCMS responsibilities were usually limited to a single individual. Also, the workforce in the surrounding region of KGH included limited DCMS experience and expertise during the course of this study.

#### Free text CRF responses

Handwritten responses in free text CRF fields were often difficult to decipher. Many of these free text entries were later replaced with closed-ended responses, but those items with over five response options (such as medication types and village of residence) were maintained in free text format to consolidate CRF space.

#### Software and equipment maintenance

Software portals and discounted academic software licenses were unavailable for DCMS personnel at KGH. While the commercial scanners and laboratory equipment were intended to undergo regular routine maintenance, it was not feasible to transport this equipment to the United States. It was therefore necessary to have multiple backup devices on hand in the event of equipment failure.

#### Internet functionality and cost

Data sharing was dependent on internet functionality, which, until recently, relied on satellite internet systems that were expensive, yielded low bandwidth, and were overburdened by the number of users. Satellite internet connectivity was often restricted to daylight working hours, where occasionally manufacturer system upgrades were carried out while the system was offline and resulted in multi-day lapses in connectivity. The transition from satellite internet systems to commercial, hard-wired systems provided improved bandwidth, but these systems were often unstable and required frequent modem resets and regular communication with the technical personnel at its main network in Sierra Leone’s capital city of Freetown. While costs for commercial hard-wired internet access were considerable, they were still less expensive than satellite internet systems.

#### Power surges

The electricity in Sierra Leone operates at 230 volts, and some of the laboratory equipment was rated for 120 volts and required step-down power transformers. Surge protectors were necessary for all electronic equipment due to frequent power surges. KGH was particularly vulnerable to electronic damage from lightning strikes, where one such strike during the course of this study resulted in the destruction of the electrical system at the KGH Lassa Ward and Laboratory and required a virtual complete rewiring of the system.

### Summary of DCMS progress

The ultimate goals of this effort were to develop and implement inexpensive, efficient, and accurate DCMS processes at KGH. The incremental progress here should be considered in the context of a low-resource environment that currently lacks the capacity for cloud-based DCMS equipped with servers that are found in more developed environments. The milestone achievements for this effort included the introduction, development, and implementation of: a DCMS serving the both hospital and research activities; a medical chart system at the KGH Lassa Ward; a laboratory blood sample tube labeling system; macro-driven processes for summarizing and consolidating data files; an electronic medical records archive; the collection and capture of blood chemistry panel data; new measureable responses for CRF items including the patient temperatures measured by thermometers; auditing processes for incorporating data for key outcomes over multiple sources; and the full linkage of Lassa fever data sources.

### DCMS literature in Sierra Leone

**Fig 5:**
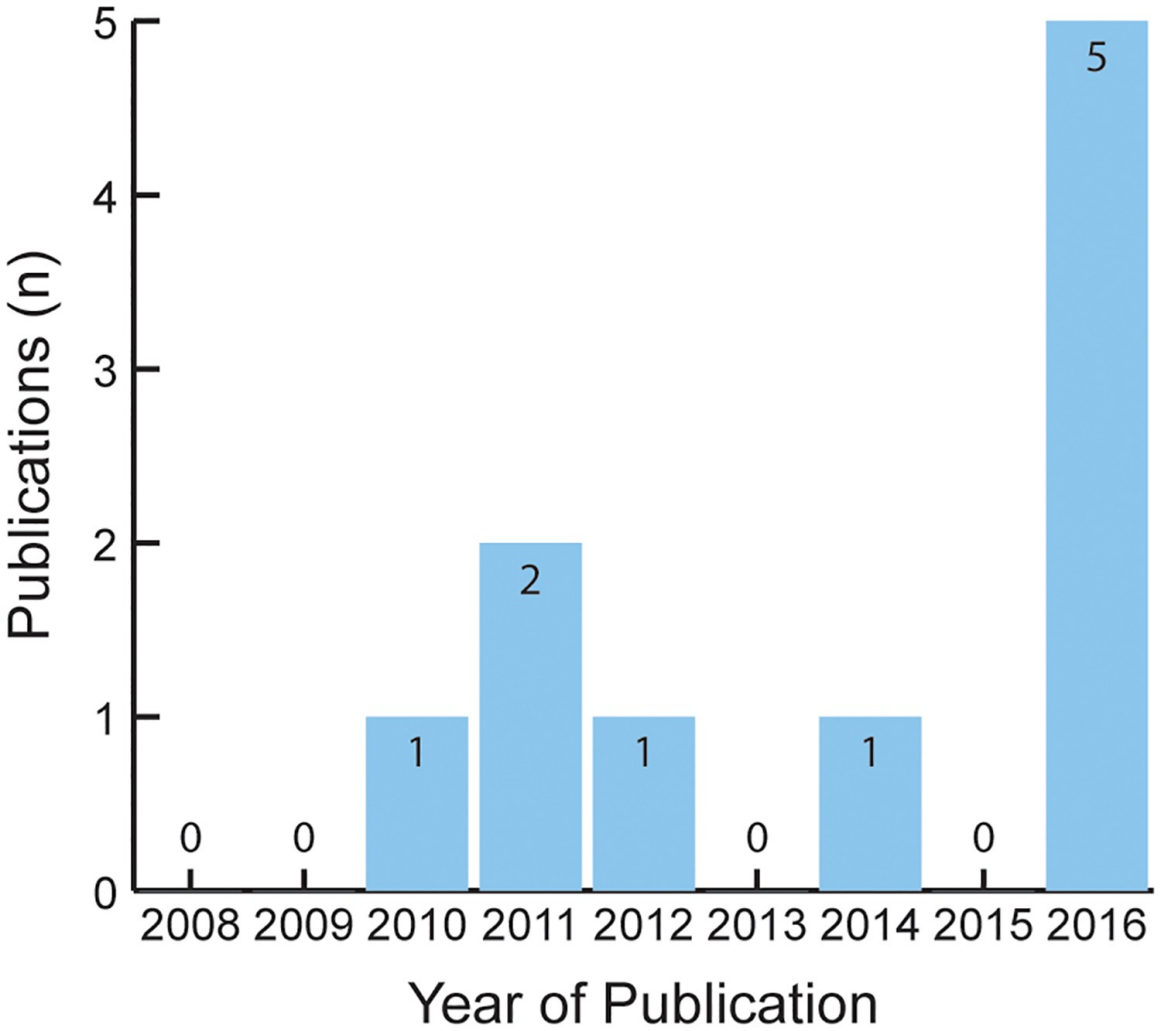
Publications related to data capture and management for Sierra Leone, 2008–2016. A bibliographic database search using the PubMed search engine produced 10 hits using key words *Sierra Leone* and *data capture* together with *data management*; *database management*; *data system(s)*; or *health information system(s)*.

Between January 2008 and December 2018, only 10 publications were observed though a bibliographic database search using the PubMed (U.S. National Library of Medicine, Bethseda, MD) search engine (key words *Sierra Leone* and *data capture* together with *data management*; *database management*; *data system[s]*; or *health information system[s]*). These publication hits are shown by year of publication in Fig 5.

The country of Denmark maintains a total population of 5,605,948 (2017 estimate), which is smaller than Sierra Leone’s total population of 6,163,195 (2017 estimate; [31]). However, repeating the PubMed search above for Denmark yielded 114 hits, which reveals the scarcity of DCMS literature for Sierra Leone. These disparities are likely attributable to Sierra Leone’s infrastructure deficiencies and weak publication rates overall.

## Discussion

We developed a DCMS for Lassa fever supporting both research and hospital activities in a limited resource hospital setting. The system is modest by international standards, but suits meets the needs of its host environment and provides a sustainable solution with a small operating budget. Several of the DCMS tasks here were fully automated through macro programing techniques, which made possible the management of thousands of data files. While the DCMS here included paper materials, the system minimized the transfer of these materials inside and outside of the laboratory. The DCMS would have considerably benefitted from a teaching computer laboratory for ongoing training purposes. While data management and infrastructure usually fall low on the hierarchy of needs in LMICs, cost sharing or fixed, long-term funding mechanisms for supporting such infrastructure is vital for building DCMS capacity building in LMIC environments. To this end, the DCMS here benefitted from being situated in a hospital setting that provided financial support for personnel and infrastructure through governmental sources. In turn, the incremental DCMS developments over the course of this effort built capacity for attracting future research endeavors and contributed to the overall infrastructural development in an area where it is greatly needed.

### Need for improved patient triaging

Weak patient triaging practices for suspected Lassa fever cases have historically overburdened DCMS and laboratory resources at KGH. While the risk of becoming infected with Lassa fever by presenting to a health center is minimal, these uninfected presentations consume valuable laboratory and hospital staff resources. Conversely, subjects infected with Lassa failing to present for screening and patient care run the risk of morbidity, mortality, or transmission. It is therefore essential to improve patient triaging practices, which is particularly challenging for Lassa due to the similarity and overlapping case definition criteria for competing illnesses such as malaria. Also, clinical signs and symptoms observed on presentation are often measured subjectively without confirmatory measures. Patient triaging may also be affected by inadequate transportation resources and seasonal rainfall. Training on proper triaging and the use of rapid diagnostic testing devices at peripheral health units are perhaps the most plausible approaches for improving triaging practices. While this work revealed weak specificity in the Lassa fever suspected case definition, additional efforts are needed to assess its sensitivity and other measures of validity prior to making wholescale changes to its construct.

### Future DCMS solutions

DCMS solutions in LMICs appear to be trending toward the utilization of mobile technologies, cloud-based data systems, and freely available, open-source software applications. Smartphone ownership is rising exponentially across Africa, and there is a critical need to take advantage of its rising use and develop more efficient, accurate approaches for outbreak response and outreach activities. Freely available software solutions such as the Research Electronic Data Capture Mobile App (REDCap, Vanderbilt, TN) may be used for disease reporting and surveillance, and applications such as MyTherapy (smartpatient, Munich, Germany) provide viable solutions for assessing and improving treatment adherence and patient follow-up. To this end, feasibility studies are needed in remote parts of Sierra Leone to determine the extent of smartphone ownership as an initial step toward their utilization in improving Lassa fever surveillance and reporting. There is also a movement towards software applications with both online and offline capabilities such as OpenClinica (OpenClinica, LLC, Waltham, MA) and REDCap [32, 33]. Quantum GIS (QGIS; Open Source Geospatial Foundation, Chicago, IL), ArcGIS Online (Esri, Redlands, CA), and R (R Foundation for Statistical Computing, Vienna, Austria) provide freely available analytical and disease mapping software solutions for low-resource environments. Commercial data management applications with annual license fees such as the SAS System may prove difficult to sustain during lapses in short-term funding. An application of particular promise and DCMS relevance is Microsoft Office365 (Microsoft, Redmond, WA), which includes cloud-based versions of its Word, Excel, and Access applications. It was recently announced that Microsoft will be delivering cloud services to customers across Africa from data centers located in South Africa [34].

### Data centers and regional thinking

Centralized DCMS serving multiple functions, sites, or entities (referred to here as “data centers”) may provide viable solutions for sustaining DCMS through cost-sharing and fixed funding sources. In our experience, such approaches are best situated in academic or health care environments where fixed funding for core infrastructure is available or feasible. Upon development, these approaches may serve as a basis for garnering new short-term research projects and increasing involvement in clinical research. Regionalizing data centers over multiple countries may further serve facilitate cost and resource sharing, but such a strategy requires considering the public health needs for its partners or neighboring countries as their own. Partnerships among short-term research personnel may also promote sustainability, and thus a national database of health research would have great utility in fostering such partnerships [17]. Modeling successful approaches found within Africa such as those in South Africa may be advantageous as its data systems are similar to those in developed countries [35, 36]. Regional partnerships may also facilitate current trends in data sharing and integration as patient-provider interactions may trend toward a focus on minimum sets of key indicators [37]. We believe that these trends will be observed in the research enterprise where DCMS will increasingly focus on limited sets of key study variables that may be standardized over research sites or multiple countries.

## Conclusions

DCMS are the backbone of research efforts in LMICs. This project was carried out in an extremely limited resource environment where ironically sophisticated DCMS are most needed. The progress here focused on building DCMS capacity by developing automated processes and responsibilities in the collection, capture, management, linkage, and archival of clinical and laboratory data. These developments were made possible by using customized DCMS processes through practical, inexpensive, and scalable solutions. The challenges and progress chronicled in this study are intended to serve and assist infectious disease researchers in designing and sustaining DCMS in similar low-resource environments. More literature on DCMS in LMICs is needed to establish benchmarks and drive goal-driven DCMS solutions and development in limited resource environments.

## Supporting information

S1 Table Individual data for Tables 3 and 4. The columns (left to right) correspond to the patient observation number, year of presentation to KGH or local peripheral health unit, time period of initial blood draw, hospital admission status, survival outcome at hospital discharge, and Lassa fever serostatus.
(XLS)
